# Evaluation of mental health and anxiety level among hepatitis C patients during COVID-19 pandemic in Pakistan

**DOI:** 10.1186/s43066-021-00120-9

**Published:** 2021-06-12

**Authors:** Sadia Rafique, Muhammad Saleem Khan, Rabia Unar, Muhammad Wajid, Ahmad Waheed, Ali Umar

**Affiliations:** 1 Department of Zoology, Faculty of Life Sciences, University of Okara, Okara, 56130 Pakistan; 2 Jinnah Post Graduate Medical Center Hospital, Karachi, Pakistan

**Keywords:** Hepatitis C, COVID-19, Anxiety, Mental health, Pakistan

## Abstract

**Background:**

This study was conducted to check anxiety level and mental stress in the 200 confirmed hepatitis C patients during the COVID-19 pandemic. The Chinese version of the Beck Anxiety Inventory (BAI) score index was used to measure the anxiety level of HCV-positive patients. BAI score index of different demographic factors such as gender, age, occupation, and education of all the sampled population was calculated.

**Results:**

The highest BAI score was recorded in people in the age group of 25–45 years (54.5%). Respondents from public sector employees, own businesses, and postgraduates were highly anxious. A significant difference in BAI score was also recorded between male and female respondents as 33.77% of females were at a severe level of anxiety compared to 17.07% of males. Furthermore, quarantined hepatitis C patients had a significantly higher BAI score (39.5) as compared to non-quarantined patients (27.12), and respondents with HCV infection also had a high BAI score of 37.25 compared to healthy individuals (4.1). Most of the respondents were willing to adopt protective measures against COVID-19.

**Conclusion:**

This study concluded that people with infectious diseases like hepatitis C had high anxiety levels and mental stress in the COVID-19 pandemic and needed psychological aids for better mental health to handle pandemic conditions.

## Background

Hepatitis is a serious health concern around the globe and especially in developing countries in Asia [1]. Hepatitis C virus (HCV) is the causative agent of hepatitis C and 3% of the world’s population is infected by this virus. Chronic hepatitis C damages hepatic tissues and, in some cases, also develops hepatic carcinoma and hepatic cirrhosis [2].

When a person is infected by HCV, the virus influences the immune system and destroys the recognition and defense system of the host body. Acute or mild infections have less impact on immunology, but if left untreated for a long period of time, the infection can progress to a chronic condition that reduces patients’ immunity. These immunosuppressed patients have more chances of developing severe complications in other diseases like COVID-19 than healthier persons [3].

COVID-19 is a pandemic viral infection, caused by a coronavirus (SARS-CoV-2). The outbreak began in Wuhan (China) and spread throughout the world [4]. To date, 151,916,040 people have been infected and 3,190,948 deaths have been reported globally due to the COVID-19 pandemic [5]. This pandemic also increases anxiety, depression, and restlessness in immune-suppressed individuals such as HCV-infected people.

Psychological problems are often observed in patients suffering from chronic HCV or undergoing HCV treatment such as riboflavin and PegIFNa (pegylated interferon-alpha). The most frequent and serious side effect of antiviral therapy is depression and poor mental health [6]. Several other factors, such as thinking of being affected by a deadly disease, the negative social impact, and higher complications, are responsible for psychological problems [7]. People are affected very badly in respect of mental health due to the COVID-19 pandemic [8], and this also increases anxiety and stress in HCV patients as recorded in this study. The lockdown situation further increases problems for chronic patients like HCV in terms of availability of medicine and fear of being infected with COVID-19 infection, and this was proved in previous studies by Guerriero et al. [9] that lockdown increases the functional neurological symptoms of mental health disorder by 3.4-fold. Furthermore, similar stressful conditions and anxiety were recorded in individuals that were in direct contact with Ebola-infected individuals (2018) in the Democratic Republic of the Congo and Sierra Leone [10]. Seeing the current situations, this study was designed to find out the anxiety level in HCV-infected individuals.

## Methods

### Sample population

A cross-sectional study was conducted in different hospitals in Punjab, Pakistan, on a random basis for 4 months. The sample size consisted of 200 HCV-infected patients, who were approached and interviewed in hospitals. Verbal consent was obtained from all individual participants before the interview and the consent was approved by the Ethical Committee, University of Okara (reference number: UO/DOZ/2020/misc.). It was made sure that the ethical standards involving human participants were followed by all means according to the ethical standards of the Ethical Committee, University of Okara, as well as the 1964 Helsinki Declaration and its later amendments or comparable ethical standards.

### Assessment of anxiety level

To evaluate the anxiety level, the Chinese version of the Beck Anxiety Inventory (BAI)-based questionnaire was used. This version was used successfully to evaluate the anxiety levels in Chinese populations [11, 12]. The BAI consisted of different questions that describe symptoms of anxiety. Respondents were asked to rate their anxiety level with symptoms on a 4-point Likert type (0–3). The total score ranged between 0 and 63 was used to rate each individual on the anxiety scale [13]. BAI scores were categorized into 4 levels. Normal respondents had scores ranging from 0 to 9, mild or moderate 10–18, moderate to severe 19–29, and severe 30–63 [14].

### Statistical analysis

The demographic profile of the sample and BAI scores were characterized by descriptive statistics. Minitab V.17 was used as a statistical tool to analyze the data.

## Results

The study population consisted of 200 confirmed HCV-infected patients that regularly visited the hospitals. Out of total respondents, 61.5% (n = 123) were male and 38.5% (n = 77) were female. Most of the respondents (n = 109, 54.5%) were between the ages of 25 and 45. Respondents were of different professions, like labor (maximum, 20.5%) and students (7.5% being minimum). The educational levels of respondents were different from undergraduate (49%), graduate (36.5%), and postgraduate (14.5%) as represented in Table 1.

**Table 1:** Demographic characteristics of HCV-positive participants

Participant characteristics	Respondents	
	*N*	%
**Gender**		
Male	123	61.5
Female	77	38.5
**Age**		
≤ 25 years	11	5.5
25–45 years	109	54.5
> 45 years	80	40
**Occupation**		
Student	15	7.5
Teachers	36	18
Unemployed	15	7.5
Agriculture	40	20
Own business	20	10
Labor workers	41	20.5
Public sector employee	33	16.5
**Education**		
Undergraduate	98	49
Graduate	73	36.5
Postgraduate	29	14.5

It was widely assumed that COVID-19 outbreaks cause anxiety and mental stress in the general population. We investigated whether this outbreak also affects patients with compromised immunity, i.e., HCV-infected patients. For this purpose, we recorded the BAI score in HCV patients. There was a significant difference in BAI scores between males and females. A high BAI score was recorded in the age group of 25–45 years, which showed severe anxiety levels in this age group in HCV-infected patients. Furthermore, we also checked whether the demographic characters influence the BAI score or not. Unlike others, a very low level of anxiety was recorded among HCV-infected students as most (80%) of students have BAI level 1.

**Fig. 1:**
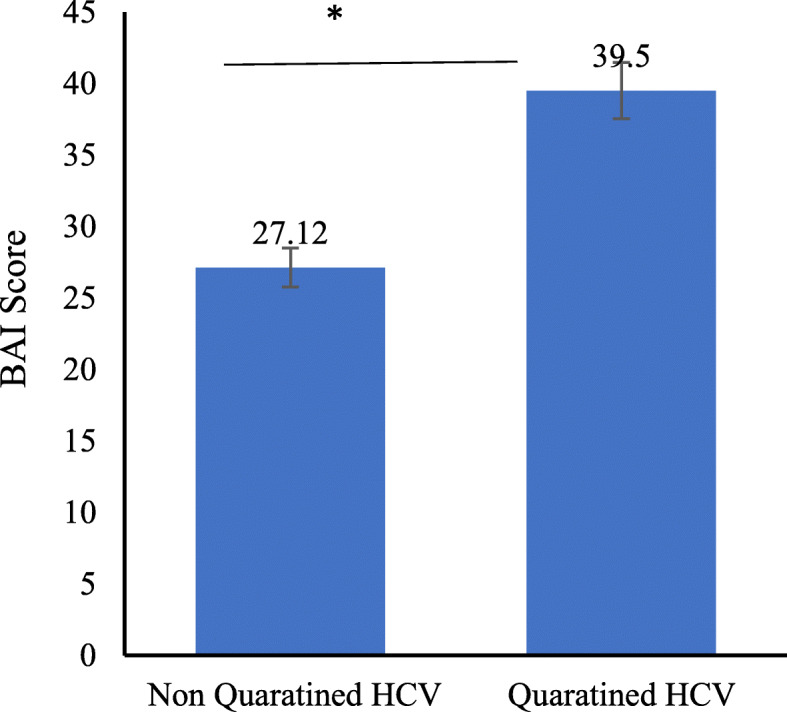
BAI score of quarantined HCV patients vs non-quarantined HCV patients

**Fig. 2:**
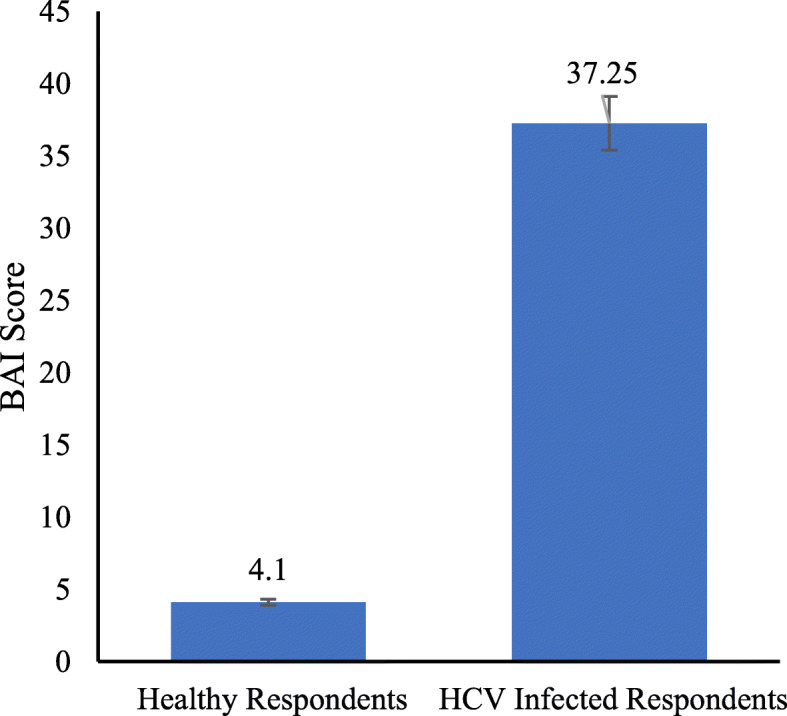
BAI score of healthy vs HCV-positive patients

The quarantined HCV patients had a significantly higher BAI score as compared to non-quarantined HCV patients (Fig. 1), while HCV-positive patients had a highly significant BAI score as compared to healthy individuals (Fig. 2).

Among the professions, the highest percentage of respondents with their business (50%) were at a severe anxiety level (30–63%). The same was in the case of postgraduate patients (Table 2).

**Table 2:** BAI score of the HCV-infected respondents classified in various subsamples

Subsamples	BAI score					
	0–9 n (%)	10–18 (%)	19–29 (%)	30–63 (%)	χ^2^	P
Gender						
Male (n = 123)	45 (36.59%)	30 (24.39%)	27 (21.95%)	21 (17.07%)	13.070	0.004
Female (n = 77)	13 (16.88%)	16 (20.78%)	22 (28.57%)	26 (33.77%)		
**Age**						
≤ 25 years (n = 11)	8 (72.73%)	2 (18.18%)	1 (9.09%)	0	13.041	0.042
25–45 years (n = 109)	29 (26.61%)	31 (28.44%)	18 (16.51%)	31 (28.44%)		
> 45 years (n = 80)	31 (38.75%)	21 (26.25%)	14 (17.5%)	14 (17.5%)		
**Occupation**						
Students (n = 15)	12 (80%)	3 (20%)	0	0	94.589	0.001
Teachers (n = 36)	4 (11.11%)	12 (33.33%)	10 (27.78%)	10 (27.78%)		
Unemployed (n = 15)	2 (13.33%)	3 (20.00%)	8 (53.33%)	2 (13.33%)		
Agriculture (n = 40)	26 (65%)	11 (27.50%)	0	3 (7.50%)		
Own business (n = 20)	2 (10%)	0	8 (40%)	10 (50%)		
Labor workers (n = 41)	12 (29.27%)	12 (29.27%)	14 (34.15%)	3 (7.32%)		
Public sector employee (n = 33)	3 (9.09%)	8 (24.24%)	10 (30.30%)	12 (36.36%)		
**Education**						
Undergraduate (n = 98)	63 (64.29%)	24 (24.49%)	6 (6.12%)	5 (5.10%)	55.165	0.002
Graduate (n = 73)	34 (46.58%)	3 (4.11%)	24 (32.88%)	12 (16.44%)		
Postgraduate (n = 29)	5 (17.24%)	9 (31.03%)	5 (17.24%)	10 (34.48%)		
**Quarantined**						
Yes (n = 32)	0	0	11 (33.37%)	21 (66.63%)	45.183	0.004
No (n = 168)	67 (39.88%)	32 (19.04%)	40 (23.81%)	29 (17.26%)		

**Fig. 3:**
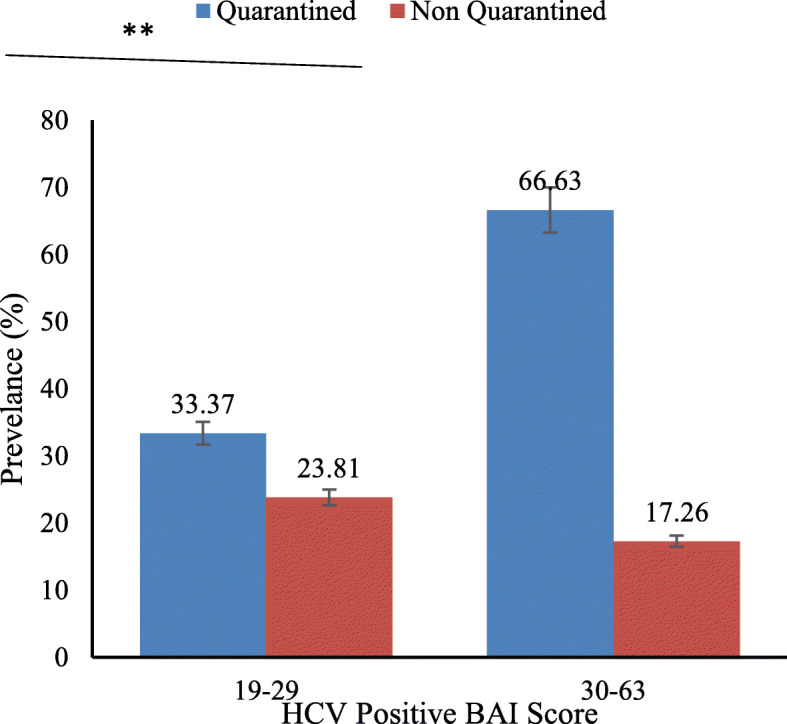
Prevalence in HCV-positive quarantined vs HCV-positive non-quarantined patients

The comparison of BAI scores of HCV-positive quarantined and HCV-positive non-quarantined patients showed that quarantined respondents had a significantly higher BAI score compared to non-quarantined patients (Fig. 3).

**Fig. 4:**
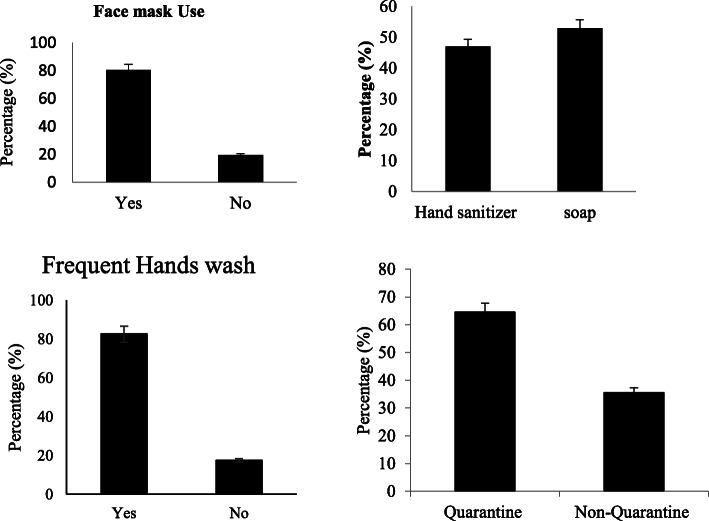
Percentage proactive measures taken by HCV-infected respondents against COVID-19 infection

Table 3 shows that most of the respondents were very conscious of adopting protective measures against being infected by COVID-19. For example, 82.5% of respondents were in the habit of using masks when they went outside their homes. The respondents were using hand sanitizers (47%), and soap (53%) for washing their hands. 82.5% of respondents were frequently washing their hands. 67% of people agreed to quarantine if symptoms of COVID-19 were experienced (Fig. 4).

**Table 3:** Prevalence of anxiety-related symptoms in HCV-infected respondents regarding COVID-19

Sr #	Parameters	Yes n (%)	No n (%)	Not sure n (%)	χ^2^	P
1	Restless due to social distancing?	109 (54.5%)	69 (34.5%)	22 (11%)	56.89	0.05
2	Significant weight loss or gain	69 (34.5%)	117 (58.5%)	14 (7%)	79.69	0.003
3	Insomnia or excessive sleep	69 (34.5%)	124 (62%)	7 (3.50%)	102.79	0.004
4	Repeated thoughts of death?	84 (42%)	105 (52.5%)	11 (5.5%)	73.03	0.013
5	Lacking interest in daily life?	123 (61.5%)	48 (24%)	29 (14.5%)	74.11	0.012
6	Fear leaving house	83 (41.5%)	107 (53.5%)	10 (5%)	76.57	0.001
7	Avoided avail healthcare facilities	116 (58%)	63 (31.5%)	21 (10.5%)	67.99	0.011
8	Limited physical contact	164 (82%)	26 (13%)	10 (5%)	215.08	0.004
9	Avoid watching the news	116 (58%)	74 (37%)	10 (5%)	85.48	0.002

## Discussion

This study aimed to record the anxiety level in HCV-positive respondents in response to the COVID-19 outbreak in the Pakistani population. It was hypothesized that the average BAI score will be higher in HCV patients compared to normal individuals. Our hypothesis was supported by the investigation of [11, 12] as their BAI score of the general population of China in an epidemic-free environment was significantly lower than the sampled population in the present study. Moreover, it was found that hepatitis C patients that were quarantined (n = 32) at the time of sampling had a higher BAI score as compared to patients that were not quarantined. These results support the view that human mental health is significantly affected by communicable diseases [15]. In fact, during the West African Ebola virus disease (EVD) outbreak, healthcare workers confirmed that, in addition to deaths and sadness, mental health services were disrupted due to the intense emotional stress faced by patients and their family members, as well as by medical professionals [16].

Out of 200 HCV-positive patients, quarantined (n = 32) patients showed a higher BAI score compared to non-quarantined most probably due to COVID-19 symptoms like sneezing, coughing, difficulty in breathing, malaise, or fever, in the quarantine period, as higher scores of depression subscale as well as Depression, Anxiety and Stress Scale (DASS) were seen in people feeling cold, sore throat, nausea, shortness of breath, and coryza in other studies [17]. Furthermore, the highest BAI score in quarantined patients could be due to viral infection, as found in previous studies, such as the association of neuropsychiatric disease with Borna virus [15], the anxiety experienced by SARS patients [18], and psychological issues such as frustration, depression, fear, anxiety, and suicidal ideation in SARS patients as well [14].

We found a significant difference in the anxiety of females and males, with more anxiety levels in females compared to males. China, Turkey, Italy, and Spain, all have reported similar results [17, 19–21]. A study by Rehman et al. [22] also recorded no significant difference in the anxiety of both sexes in the COVID-19 pandemic. The respondents in the age group of 25–45 years showed higher anxiety levels as compared to the youngsters (≤ 25 years) and older people (> 45 years), and this might be due to 25–45-year-old participants are the backbone of their family and they have more burdens of life as well as responsibilities compared to other age groups. Moreover, older people have had more severe anxiety (17.5%) as compared to younger ones. This could be due to a fear of death and dying as a result of an unknown disease. Our study differed in this aspect from Shah et al. [23] and Ozamiz-Etxebarria et al. [24] who found elevated levels of anxiety in age groups 18–24 and 18–26 respectively.

In a comparison based on occupation, those who had owned a business were more anxious (50%) than all other categories, followed by those who worked in the public sector (36.36%). Students found normal (80%) than teachers (11.11%). These outcomes were also opposed to the study of Shah et al. [23] and Cao et al. [25] who found higher levels of anxiety in students, while Rehman et al. [22] found a moderate level of anxiety in students, researchers, and health professionals in India. Furthermore, postgraduates also had anxiety levels (34.48%) followed by graduates (16.44%) and undergraduates (5.10%). This finding contradicted the findings of Zhao et al. [26] who discovered that people in high school or below experienced severe anxiety in China due to COVID-19.

## Conclusion

This study concluded that people with infectious diseases such as hepatitis C have higher anxiety levels during COVID-19 pandemic conditions and require adequate psychological aids for better mental health.

## Data Availability

Not applicable.

## Abbreviations

BAI: Beck Anxiety Inventory
SARS-CoV-2: Severe acute respiratory syndrome coronavirus 2
DASS: Depression, Anxiety and Stress Scale
EVD: Ebola virus disease
HCV: Hepatitis C virus
MERS: Middle East respiratory syndrome
SARS: Severe acute respiratory disease
